# Altered Expiratory Flow Dynamics at Peak Exercise in Adult Men With Well-Controlled Type 1 Diabetes

**DOI:** 10.3389/fphys.2022.836814

**Published:** 2022-02-18

**Authors:** Vesa V. Hyrylä, Antti-Pekka E. Rissanen, Juha E. Peltonen, Anne S. Koponen, Heikki O. Tikkanen, Mika P. Tarvainen

**Affiliations:** ^1^Department of Applied Physics, University of Eastern Finland, Kuopio, Finland; ^2^Department of Sports and Exercise Medicine, Clinicum, University of Helsinki, Helsinki, Finland; ^3^HULA—Helsinki Sports and Exercise Medicine Clinic, Foundation for Sports and Exercise Medicine, Helsinki, Finland; ^4^School of Medicine, Institute of Biomedicine, University of Eastern Finland, Kuopio, Finland; ^5^Department of Clinical Physiology and Nuclear Medicine, Kuopio University Hospital, Kuopio, Finland

**Keywords:** cardiopulmonary exercise test, elastic recoil, principal component analysis—PCA, pulmonary function, ventilatory flow

## Abstract

Type 1 diabetes may, in time, cause lung dysfunction including airflow limitation. We hypothesized that ventilatory flow morphology during a cardiopulmonary exercise test (CPET) would be altered in adult men with well-controlled type 1 diabetes. Thirteen men with type 1 diabetes [glycated hemoglobin A_1c_ 59 (9) mmol/mol or 7.5 (0.8)%, duration of diabetes 12 (9) years, and age 33.9 (6.6) years] without diagnosed diabetes-related complications and 13 healthy male controls [age 37.2 (8.6) years] underwent CPET on a cycle ergometer (40 W increments every 3 min until volitional fatigue). We used a principal component analysis based method to quantify ventilatory flow dynamics throughout the CPET protocol. Last minute of each increment, peak exercise, and recovery were examined using linear mixed models, which accounted for relative peak oxygen uptake and minute ventilation. The type 1 diabetes participants had lower expiratory peak flow (*P* = 0.008) and attenuated slope from expiration onset to expiratory peak flow (*P* = 0.012) at peak exercise when compared with the healthy controls. Instead, during submaximal exercise and recovery, the type 1 diabetes participants possessed similar ventilatory flow dynamics to that of the healthy controls. In conclusion, men with relatively well-controlled type 1 diabetes and without clinical evidence of diabetes-related complications exhibited attenuated expiratory flow at peak exercise independently of peak oxygen uptake and minute ventilation. This study demonstrates that acute exercise reveals alterations in ventilatory function in men with type 1 diabetes but not until peak exercise.

## Introduction

Type 1 diabetes has been linked to pulmonary dysfunction (Kolahian et al., 2019), which is typically absent at the time diabetes is diagnosed but develops over time. Understanding pathophysiological mechanisms underlying diabetes-related pulmonary manifestations is important when outlining their potential clinical significance and implications (Kolahian et al., 2019; Kopf et al., 2021).

Reported signs of type 1 diabetes-related pulmonary dysfunction include restricted lung capacity (Niranjan et al., 1997), reduced lung elastic recoil (Schuyler et al., 1976), reduced dynamic lung compliance (Strojek et al., 1992), peripheral airway obstruction (Mancini et al., 1999), and diminished lung diffusion capacity (Wheatley et al., 2011). In addition, lower respiratory muscle strength has been linked to diabetes (Fuso et al., 2012) and reduced maximal minute ventilation to type 1 diabetes (Komatsu et al., 2005; Koponen et al., 2013). Nevertheless, lung dysfunction in type 1 diabetes has tended to be overlooked, as it does not seem to hinder normal daily activities (Goldman, 2003). Patients with type 1 diabetes have also exhibited reduced peak pulmonary oxygen uptake (V̇O_2peak_) (Komatsu et al., 2005; Peltonen et al., 2012; Rissanen et al., 2015), which is an integrated measure of pulmonary, cardiovascular, and skeletal muscle function (Wagner, 1996) and thus a strong predictor of respiratory and cardiovascular morbidity and mortality (Steell et al., 2019).

The aim of this study was to assess ventilatory function during a cardiopulmonary exercise test (CPET) to reveal early diabetes-related pulmonary complications in men with well-controlled type 1 diabetes. Specifically, we used a principal component analysis based method to determine ventilatory dynamics throughout the exercise protocol. While diabetes may affect the lung by several mechanisms over time, it is unknown if ventilatory flow adjustments to different intensities of acute dynamic exercise are affected already at early stages of the disease.

## Materials and Methods

This retrospective and cross-sectional study was a part of an EDGE (Exercise, Diet and Genes in Type 1 Diabetes Mellitus) study belonging to a Canadian-Finnish research collaboration entitled “ARTEMIS—Innovation to Reduce Cardiovascular Complications of Diabetes at the Intersection of Discovery, Prevention and Knowledge Exchange” (Noble et al., 2013). Twenty-six non-smoking, normally physically active males, including 13 type 1 diabetes participants (DM) and 13 healthy controls matched for peak minute ventilation (CON), underwent a CPET until volitional exhaustion in a previously described clinical laboratory setting (see Rissanen et al., 2018 for further details). The DM group had no diagnosed or evidence of diabetes-related microvascular complications (neuropathy, nephropathy, and retinopathy), hypertension, or any chronic diseases expect for their type 1 diabetes. Furthermore, the participants were under no medication apart from insulin therapy of type 1 diabetes participants. The CON participants had no long-term or chronic diseases and were overall healthy. Although all the subjects were non-smoking during the time of study, three control participants had a smoking background or had been casually smoking years before the study. The Ethics Committee of the Hospital District of Helsinki and Uusimaa, Helsinki, Finland approved the study (308/13/03/00/2008, September 2, 2008). The participants gave their written informed consent prior to participating the study.

Physical activity was determined from a questionnaire. The participants were asked to record how many times per week and for how long per time they usually exercise with moderate or vigorous intensity. Total exercise time per week was then summarized and regarded as individual’s self-reported physical activity. Participants underwent flow-volume spirometry (Medikro Spiro 2000, Medikro, Kuopio, Finland and z-scores according to Kainu et al., 2016) measurements at rest and a CPET under laboratory conditions on a cycle ergometer (Monark Ergomedic 839E; Monark Exercise AB, Vansbro, Sweden). A 12-lead electrocardiogram was recorded using Powerlab system (ADInstruments, Oxford, United Kingdom). Ventilatory flow was measured throughout the CPET with a low resistance turbine (Triple V; Jaegar Mijnhardt, Bunnik, Netherlands), which was calibrated with a syringe (3.00 L, Hans Rudolph Inc., Kansas City, MO, United States). The sampling frequency of the ventilatory flow was 33.33 Hz, which is adequate for capturing ventilatory flow characteristics as the meaningful frequency content is below 10 Hz. Later on, the flow data were upsampled to 100 Hz to achieve 0.01 s point precision for the determination of ventilatory flow cycles. Pulmonary oxygen uptake was measured continuously (AMIS 2000; Innovision A/S, Odense, Denmark).

The CPET measurement protocol consisted of a 5-min rest on the ergometer followed by a 5-min warm-up (unloaded pedaling). After the warm-up, the exercise protocol started with a 40 W load and the work rate was increased by 40 W every 3 min until volitional fatigue (peak exercise). Finally, a recovery period (sitting on the ergometer) of 5 min followed the peak exercise. V̇O_2peak_ and the highest point of end-tidal *P*_CO_2__ and estimated arterial *P*_CO_2__ (Jones et al., 1979) were determined as the maximum value of moving average filtered data (30-s window width). In addition, end-tidal *P*_CO_2__ and estimated arterial *P*_CO_2__ were assessed from the last 30 s of exercise (peak exercise). V̇O_2_/work rate slope was determined from the onset of incremental exercise until volitional exhaustion. Ventilatory threshold 1 was primarily determined by the V-slope method (i.e., identifying a consistent increase in the CO_2_ output/V̇O_2_ relationship) (Beaver et al., 1986). Minute ventilation/CO_2_ output slope (V̇E/V̇CO_2_ slope) was determined from the onset of incremental exercise until a respiratory compensation point (Neder et al., 2017); the respiratory compensation point was primarily determined by identifying a consistent increase in the V̇E/V̇CO_2_ relationship (Beaver et al., 1986). V̇O_2peak_ of predicted (%) was determined based on Hansen et al. (1984) as recommended (Guazzi et al., 2012). Breathing rate was determined from the continuous airflow using Welch’s periodogram with three 50% overlapping Hanning windows, and minute ventilation was calculated using breathing rate and expiratory tidal volume.

The CPET data analysis was conducted in separate windows from the beginning of the exercise to the recovery. One-minute analysis windows were placed to the end of every fully completed exercise step. In addition, 1-min windows were placed at subjective peak exercise (last minute before fatigue) and to the last minute of the recovery period. These choices allow comprehensive evaluation of the ventilatory flow from the beginning of exercise to the recovery while ensuring robustness.

### Ventilatory Flow Analysis

**FIGURE 1:**
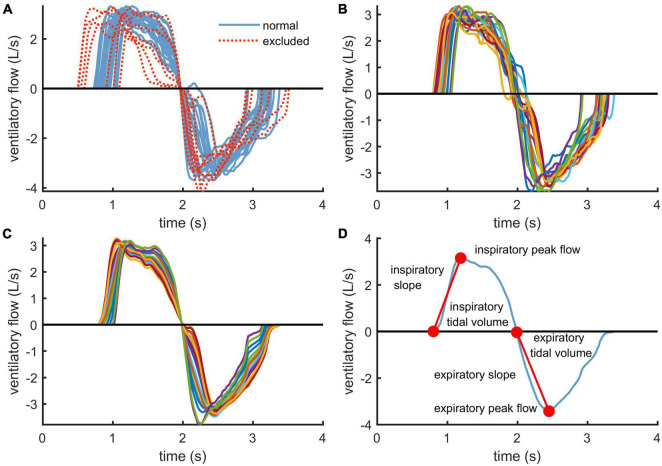
Clarification of the ventilatory flow analysis workflow: **(A)** exclusion of extreme flow cycles within an ensemble using L1-norm, **(B)** time-aligned ensemble using the genetic algorithm, **(C)** principal component transformed flow cycles and **(D)** derivation of the dynamic ventilatory flow parameters from each flow cycle.

In this section, we propose a robust analysis method for the ventilatory flow as flow characteristics can vary substantially at lower exercise intensities and is subject to gasping, coughing, and swallowing, for example. To capture subjective ventilatory dynamics, inspiratory and expiratory phases were identified with an in-house zero-crossing algorithm. A consecutive inspiratory phase followed by an expiratory phase constituted a single flow cycle. Flow cycles within an analysis window formed an ensemble, in which flow cycles were aligned according to expiration onset time. As breathing tends to vary, 25% of flow cycles were excluded by computing L1-norm of z-score standardized parameters. These standardized parameters were inspiration onset, inspiratory peak flow, time of the inspiratory peak flow, end of expiration, expiratory peak flow, and time of the expiratory peak flow. The top 25% of cycles, which received the highest norm values, were excluded as divergent cycles. An example of excluded flow cycles within one ensemble is presented in Figure 1A.

As time-alignment of flow cycles is crucial in an ensemble analysis, we improved alignment of the cycles by using a genetic algorithm. Our genetic algorithm searches for optimally aligned ensemble using a fitness function that is based on the principal component analysis. This method decomposes a set of correlated flow cycles into a set of uncorrelated principal components and their eigenvalues, which describe the amount of variation a single principal component can explain. The fitness function used in the genetic algorithm calculates the ratio of the first (largest) eigenvalue with respect to the rest of the eigenvalues, which is maximized when the first principal component fits the ensemble data optimally. This ratio has been proposed as an option for time-alignment previously by Garde et al. (2017).

The genetic algorithm provides a sub-optimal solution, which improves alignment by allowing cycles to be shifted not more than half a second forward or backward. Once sub-optimal alignment has been achieved (Figure 1B), flow cycles are denoised by estimating each cycle with two or more principal components that explain at least 95% of the flow cycle ensemble variance (Figure 1C). Principal components corresponding to the smallest eigenvalues are treated as noise, and thus, their contribution to flow cycles is filtered out. Principal component analysis has previously been used with flow cycles to distinguish abnormal respiratory morphologies in patients with chronic heart failure (Garde et al., 2011).

Ventilatory flow parameters were first computed for every flow cycle separately and then averaged over the ensemble to obtain mean values reflecting properties of an average cycle. Determined parameters were ventilatory tidal volume, peak flow, and slope for both inspiratory and expiratory phases of the ventilatory cycle (see Figure 1D). Tidal volume is the volume of air moved into or out of the lungs during inspiration or expiration during a single respiratory flow cycle. Peak flow captures the maximal airflow produced inward or outward, which depends on elastic properties of the lung, resistance of the airways, and respiratory muscle activation. Finally, pulmonary capacity to accelerate airflow is quantified by the slope, which connects start of each ventilatory phase to the peak flow point.

### Statistics

Variables are presented as mean (SD) or as mean (95% CI). Demographics, gas exchange, and spirometry parameters were compared with Welch two sample *t*-test. Longitudinal analysis setting is prone to missing data points and induces correlation due to repeated measurements. To account for these circumstances, we used linear random intercept models with fixed effects of group, load, minute ventilation, interaction between load and group as well as interaction between load and minute ventilation. In addition, we included relative V̇O_2peak_ as a covariate and treated load as a categorical variable. We used restricted maximum likelihood estimation for fitting and estimated marginal means for stepwise group comparison with Kenward-Roger degrees of freedom approximation. Statistical analysis was only considered at the points where both of the groups had seven or more subjects. Expiratory peak flow, inspiratory slope, and expiratory slope were log-transformed to meet multivariate normality assumptions. Lastly, we fitted a linear model to compare the associations between minute ventilation and expiratory peak flow and expiratory slope at peak exercise between groups. Predictors of the linear model were: group, peak ventilation, and interaction between group and peak ventilation. Peak ventilation was zero-centered before the fit; thus, the intercept describes the within-group value of an expiratory flow parameter at mean peak ventilation. Statistical analyses were conducted with R 4.0.2 (R Core Team, 2020).

## Results

DM and CON groups were comparable in terms of age, physical activity, anthropometrics, and resting blood pressure, as presented in Table 1. Moreover, forced expiratory volume in 1 s (FEV1), forced vital capacity (FVC), and FEV1/FVC were similar between the groups.

**TABLE 1:** Descriptive statistics.

	DM (*n* = 13)	CON (*n* = 13)	*P*-value
Age (y)	33.9 (6.6)	37.2 (8.6)	0.283
Self-reported physical activity (h:min/wk)	2:32 (2:05)	3:24 (2:00)	0.299
Body mass (kg)	78.4 (11.2)	79.8 (6.7)	0.700
BMI (kg/m^2^)	24.8 (3.0)	24.8 (2.1)	0.995
Height (cm)	178 (8)	179 (3)	0.483
FEV1 (L)	4.47 (0.50)	4.43 (0.60)	0.837
FEV1 (z-score)	0.10 (1.10)	0.00 (1.16)	0.830
FVC (L)	5.36 (0.55)	5.43 (0.71)	0.774
FVC (z-score)	-0.28 (0.92)	-0.21 (1.20)	0.869
FEV1/FVC (%)	84.24 (6.71)	81.35 (5.56)	0.243
FEV1/FVC (z-score)	0.65 (1.07)	0.29 (1.06)	0.401
Systolic blood pressure at rest (mmHg)	131 (14)	132 (22)^†^	0.899
Diastolic blood pressure at rest (mmHg)	83 (8)	81 (16)^†^	0.674
HbA_1c_ (%)	7.5 (0.8)^†^		
HbA_1c_ (mmol/mol)	59 (9)^†^		
DM duration (y)	12 (7)		

*^†^Stands for n = 12; variables are presented as mean (SD); DM, diabetes group; CON, control group; HbA_1c_, glycated hemoglobin A_1c_.*

Table 2 summarizes CPET data. The participants’ efforts during CPET were similarly maximal in the DM and CON groups based on respiratory exchange ratio, rating of perceived exertion, and % of age-predicted maximal heart rate at peak exercise. The DM group was unable to reach as high maximal work rate as the CON group. V̇O_2_/work rate slope was normal and equal in DM and CON (9.9 ± 1.0 mL/min/W vs. 9.9 ± 0.8 mL/min/W, respectively, *P* = 0.948). Due to observing moderate, albeit statistically insignificant, between-group differences in V̇O_2peak_ (absolute L/min: *P* = 0.060; relative mL/kg/min *P* = 0.107), we included the relative V̇O_2peak_ as a covariate in the linear mixed models to account for this difference. When interpreted as % of predicted, V̇O_2peak_ was lower in DM than in CON. At peak exercise, minute ventilation, breathing rate, end-tidal *P*_CO_2__, and estimated arterial *P*_CO_2__ did not differ between DM and CON. Instead, arterial O_2_ saturation at peak exercise was statistically lower in CON than in DM but within or very close to normal levels. V̇O_2_ at ventilatory threshold 1 did not differ between DM and CON. In addition, V̇E/V̇CO_2_ slopes reflected ventilatory efficiency to be both normal and comparable between the groups, and also the highest values of end-tidal and estimated arterial *P*_CO_2__ were similar between the groups.

**TABLE 2:** Cardiopulmonary exercise test.

	DM (*n* = 13)	CON (*n* = 13)	*P*-value
**Peak exercise**			
Work rate (W)	228 (34)	260 (37)	0.028
V̇O_2peak_ (L/min)	2.78 (0.46)	3.13 (0.45)	0.060
V̇O_2peak_ (mL/kg/min)	35.6 (4.5)	39.8 (7.6)	0.107
V̇O_2peak_ of predicted (%)	94 (14)	107 (15)	0.029
Heart rate (bpm)	182 (11)	177 (12)	0.255
Heart rate of age-predicted maximum (%)	98 (5)	97 (6)	0.623
Respiratory exchange ratio	1.22 (0.07)	1.18 (0.04)	0.101
Rating of perceived exertion	19 (1)	19 (1)	0.547
SpO_2_ (%)	97 (2)	95 (3)	0.011
End-tidal *P*_CO_2__ (mmHg)	36 (6)	36 (5)	0.715
Estimated arterial *P*_CO_2__ (mmHg)	38 (5)	38 (5)^†^	0.852
Minute ventilation (L/min)	113 (24)	121 (14)	0.321
Breathing rate (breaths/min)	44 (7)	44 (8)	0.899
Inspiratory time (s)	0.71 (0.10)	0.71 (0.12)	0.938
Expiratory time (s)	0.80 (0.13)	0.72 (0.11)	0.114
Mean inspiratory flow (L/s)	3.4 (0.7)	3.7 (0.4)	0.251
Mean expiratory flow (L/s)	3.4 (0.8)	4.0 (0.5)	0.047
**Ventilatory threshold 1**			
V̇O_2_ (L/min)	1.83 (0.41)	1.88 (0.32)	0.714
V̇O_2_ (mL/kg/min)	23.4 (4.7)	23.9 (5.5)	0.781
**Ventilatory efficiency**			
V̇E/V̇CO_2_ slope	26 (3)	28 (4)	0.186
Highest end-tidal *P*_CO_2__ (mmHg)	49 (3)	49 (5)	0.852
Highest estimated arterial *P*_CO_2__ (mmHg)	50 (3)	50 (4)^†^	0.778

*^†^Stands for n = 12; variables are presented as mean (SD); DM, diabetes group; CON, control group.*

**FIGURE 2:**
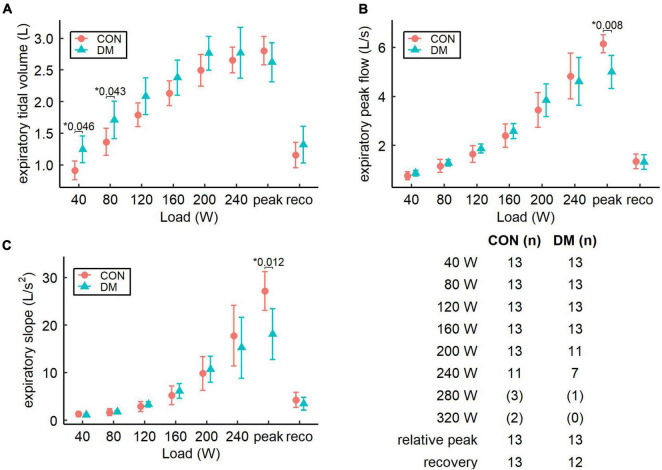
Pairwise comparison of estimated expiratory flow parameters (mean and 95% CI): **(A)** expiratory tidal volume, **(B)** expiratory peak flow, and **(C)** expiratory slope for the control (CON) and the diabetes (DM) groups. Table describes the number of participants for both groups and for each increment. Increments with less than seven subjects are not shown in the graphs and the sample sizes for these increments are presented in parenthesis. * Only *P*-values less than 0.05 are shown.

**FIGURE 3:**
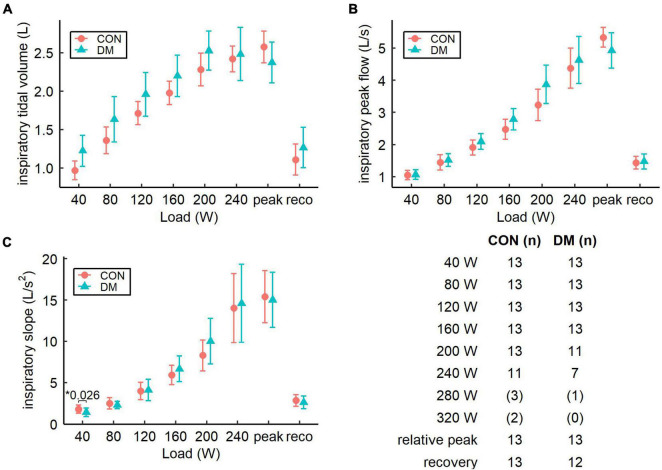
Pairwise comparison of estimated inspiratory flow parameters (mean and 95% CI): **(A)** inspiratory tidal volume, **(B)** inspiratory peak flow, and **(C)** inspiratory slope for the control (CON) and the diabetes (DM) groups. Table describes the number of participants for both groups and for each increment. Increments with less than seven subjects are not shown in the graphs and the sample sizes for these increments are presented in parenthesis. *Only *P*-values less than 0.05 are shown.

Parameters related to ventilatory flow dynamics are presented for the expiratory phase in Figure 2 and for the inspiratory phase in Figure 3. At peak exercise, the DM group had lower expiratory peak flow (*P* = 0.008) and attenuated expiratory slope (*P* = 0.012) when compared with the CON group (Figure 2). In addition, mean expiratory flow at peak exercise was lower in the DM group as compared to the CON group (Table 2). Furthermore, the DM group had higher expiratory tidal volume at 40 W and 80 W loads (*P* = 0.046 and *P* = 0.043, respectively, Figure 2), and declined inspiratory slope at 40 W load (*P* = 0.026, Figure 3) compared to the CON group. During recovery, the groups were comparable for both inspiratory and expiratory phases.

**FIGURE 4:**
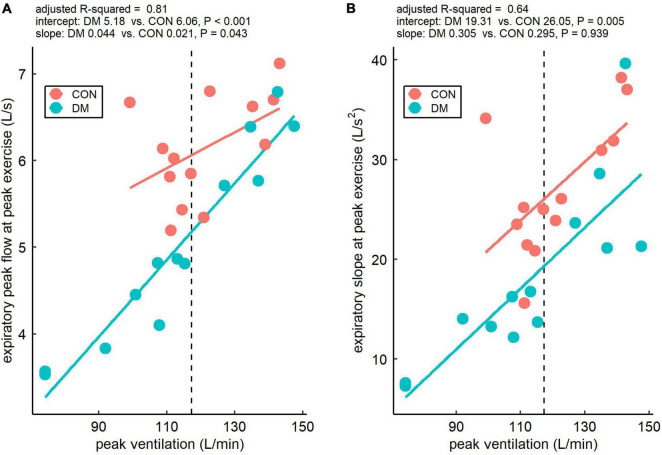
Relationship of expiratory peak flow **(A)** and expiratory slope **(B)** with minute ventilation at peak exercise for the control (CON) and the diabetes (DM) groups. The dashed vertical lines represent the mean of peak ventilation within the study participants, and a linear model was fitted to the data to compare intercepts and slopes between the groups. The intercepts describe the absolute within-group values of the two expiratory flow parameters at the mean peak ventilation (117 L/min) and the slopes describe the effect of ventilation on the two expiratory flow parameters within the groups.

Finally, the associations between minute ventilation and expiratory peak flow and expiratory slope at peak exercise are presented in Figure 4. Linear dependency between peak minute ventilation and both expiratory parameters is clearly observed. However, at the mean peak minute ventilation, the intercepts of the DM group were lower for expiratory peak flow (*P* < 0.001) and expiratory slope (*P* = 0.005) compared to the CON group.

## Discussion

The results of this study indicate altered ventilatory flow dynamics in men with relatively well-controlled type 1 diabetes at peak exercise but not during submaximal exercise. In particular, the DM group had lower expiratory peak flow and declined expiratory slope at peak exercise when compared with the CON group. Importantly, we accounted for minute ventilation and relative V̇O_2peak_ in the linear mixed models. Thus, the observed alterations in expiratory flow dynamics are rather linked with type 1 diabetes than due to any between-group differences in minute ventilation or V̇O_2peak_. This is also supported by Figure 4, which shows that expiratory peak flow and expiratory slope at peak exercise were lower in the DM group than in the CON group at a given level of minute ventilation at peak exercise. Based on the findings, at peak exercise, individuals with well-controlled type 1 diabetes require more time to produce their expiratory peak flow and eventually also attain lower expiratory peak flow compared to healthy individuals. These findings suggest that type 1 diabetes, even when it is relatively well-controlled, targets the lung, but the observed diabetes-related pulmonary manifestations are only observable at peak ventilatory demands and remain subclinical during normal daily activities.

Mechanisms behind the observed alterations in expiratory flow dynamics in type 1 diabetes may involve diabetes-related reduction of lung elastic recoil (Schuyler et al., 1976), which mimics accelerated aging (Hsia and Raskin, 2007). Reduced elastic recoil reduces the alveolar-intrapleural driving pressure of expiratory airflow and may thus exaggerate dynamic airway compression, which is magnified further if other mechanisms limiting airflow also exist (West, 2012). In terms of other coexisting mechanisms limiting expiratory airflow, signs of peripheral airway obstruction, not necessarily detected by routine pulmonary function tests at rest (e.g., flow-volume spirometry), have been reported in adults with type 1 diabetes (Mancini et al., 1999). In addition to reduced elastic recoil, reduced dynamic lung compliance has been observed in patients with type 1 diabetes and hypothesized to be another sign of diminished lung elasticity (Strojek et al., 1992). Such diabetes-related signs of pulmonary dysfunction are associated with hyperglycemia-induced pathological processes targeting the lung structure: experiments in lungs of rats with streptozotocin-induced diabetes have revealed accumulation of elastin and collagen with reduced breakdown of connective tissue protein structures (Ofulue and Thurlbeck, 1988) and recently also substantial impairments in respiratory mechanics (e.g., increased lung tissue viscance and elastance) linked to morphological and biochemical hyperglycemia-related alterations (e.g., alveolar septal thickening, alveolar collapse, inflammatory cellular infiltration) (Machado et al., 2021). In humans, reduced total lung capacity as an indicator of lung volume restriction has been found in hyperglycemic but not in normoglycemic adults with type 1 diabetes (Niranjan et al., 1997), which further suggests glycemic status is linked with lung structure and function. In addition, studies utilizing non-invasive methodology have suggested that diabetes might limit the functional reserves of the pulmonary vasculature (Chance et al., 2008; Roberts et al., 2018), which has the largest microvascular bed in the body. However, potential effects of diabetes-related pulmonary microvascular disease on lung structure and function are unclear.

One further mechanism potentially explaining the diabetes-related alterations in expiratory flow dynamics is linked to autonomic nervous system. Existing diabetic autonomic neuropathy has been shown to affect bronchomotor tone and airway caliber at rest and during tilt testing (Santos e Fonseca et al., 1992), and it has been hypothesized that dysregulated bronchomotor tone might exaggerate airway stiffness during exercise in patients with diabetes (Nesti et al., 2020). If existing, such exaggerated airway stiffness during exercise would magnify exertional dynamic airway compression and expiratory airflow limitation (West, 2012). However, the patients belonging to the DM group in our study had no history or clinically overt symptoms or signs of autonomic neuropathy. In addition, CPET unmasked no obvious signs of autonomic dysfunction in the DM group, when it comes to heart rate responses, ventilatory efficiency (reflected by V̇E/V̇CO_2_ slope), or the highest values (i.e., the setpoints) of both end-tidal and estimated arterial *P*_CO_2__, for instance. Thus, our data do not support autonomic dysfunction to be a likely mechanism behind the diabetes-related alterations in expiratory flow dynamics in this study.

The observed alterations in expiratory flow dynamics in the DM group have potential to affect tolerance of acute stress such as acute dynamic exercise. The reduced expiratory parameters in the DM group at peak exercise reflect expiratory flow limitation and may lead to excessive work of breathing at least near-maximal exercise intensities. This might have widespread implications for respiratory and skeletal muscle fatigue and thereby exercise tolerance as increased work of breathing has potential to affect systemic blood flow distribution along with increasing exercise intensity as extensively reviewed by Sheel et al. (2018). Niranjan et al. (1997) previously measured ventilatory power requirement, linked to work of breathing, and found it to be exaggerated in adults with type 1 diabetes from low-to-maximal exercise intensities. A greater demand for ventilatory power together with diabetes-related reductions in respiratory muscle strength and endurance (Fuso et al., 2012) might promote exercise intolerance in type 1 diabetes. We did observe elevated expiratory tidal volume at 40 W and 80 W and declined inspiratory slope at 40 W load in the DM group, probably suggesting a greater ventilatory demand at low-to-moderate loads. However, this is not likely due to pathological effects of diabetes but rather a consequence of higher relative work rate at fixed loads as the DM group had lower maximal work rate. Moreover, we measured neither work of breathing nor respiratory muscle characteristics in our current study; thus, this study provides no evidence of potential diabetes-related increase in work of breathing or its detrimental effects on exercise tolerance.

Although they may have only minor implications for tolerance of everyday activities and stress in relatively young adults with well-controlled type 1 diabetes, our findings of the diabetes-related alterations in expiratory flow dynamics may on a large scale be regarded as an early and subclinical sign of diminished respiratory reserves. Indeed, patients with diabetes often have diminished respiratory and/or cardiovascular reserves, which may be unmasked by acute exercise provocations (e.g., CPET possibly combined with multimodal imaging data) and develop via several multi-organ mechanisms as comprehensively reviewed elsewhere (Poitras et al., 2018; Nesti et al., 2020; Pugliese et al., 2021). From a perspective beyond exercise provocations, diminished respiratory reserves of patients with diabetes may become unmasked also when they are challenged by acute illness, which has been proposed to be the case during the ongoing SARS-CoV-2 pandemic (Caruso and Giorgino, 2020). Physical activity and exercise are an important part of overall management of diabetes (Colberg et al., 2016) and also associated with reduced mortality in type 1 diabetes (Tikkanen-Dolenc et al., 2017). As regards lung structure and function, a recent study on rats with streptozotocin-induced diabetes demonstrated how performing moderate-intensity physical exercise efficiently protected against diabetes-induced alterations in lung histology and mechanics (Machado et al., 2021). However, further basic, translational, and clinical research on the mechanisms, predictors, modifiers, and natural course of diabetes-related pulmonary manifestations are needed to identify the clinical significance of and optimal ways to prevent and treat the “diabetic lung” (Kolahian et al., 2019; Kopf et al., 2021).

### Strengths and Limitations

The strengths of this study include the data collection itself, where high-quality ventilatory flow was recorded throughout the CPET protocol. Furthermore, a new approach using principal component analysis together with the genetic algorithm was proposed for the analysis of ventilatory flow dynamics. In contrast, this study is limited by a relatively small sample size, in addition to which the results apply only to male sex. It should also be noted that timing of the volitional fatigue with respect to the exercise protocol work rate increments varied between participants, but the timing was similar between the DM and CON groups. In addition, the methodology used for flow cycle estimation effectively excluded divergent flow cycles, thus providing representative flow cycle estimates, regardless of the timing of the volitional fatigue.

## Conclusion

In conclusion, our results indicate altered expiratory flow dynamics in men with relatively well-controlled type 1 diabetes. This was observed as reduced expiratory peak flow and attenuated expiratory slope from expiration onset to the expiratory peak flow at peak exercise near V̇O_2peak_ in the DM group. However, such diabetes-related pulmonary manifestations were not observed at low-to-moderate ventilatory demands. Overall, these novel findings emphasize the lung as a target organ in diabetes mellitus but suggest that diabetes-related pulmonary complications at early stage of type 1 diabetes are subclinical and hardly affect ventilatory function during most of the daily activities.

## Data Availability Statement

The datasets presented in this article are not readily available due to privacy statements. Requests to access the datasets should be directed to the corresponding author.

## Ethics Statement

The studies involving human participants were reviewed and approved by the Ethics Committee of the Hospital District of Helsinki and Uusimaa, Helsinki, Finland. The patients/participants provided their written informed consent to participate in this study.

## Author Contributions

VH analyzed the data and drafted the manuscript. A-PR, AK, and JP collected the data. HT and JP devised the study and acquired funding for the study. MT devised analyses and acquired funding. All authors revised manuscript critically for important intellectual content, approved the final version of the manuscript, and agreed to be accountable for all the aspects of work.

## Conflict of Interest

The authors declare that the research was conducted in the absence of any commercial or financial relationships that could be construed as a potential conflict of interest.

## Publisher’s Note

All claims expressed in this article are solely those of the authors and do not necessarily represent those of their affiliated organizations, or those of the publisher, the editors and the reviewers. Any product that may be evaluated in this article, or claim that may be made by its manufacturer, is not guaranteed or endorsed by the publisher.
